# Linking Agricultural Activity Frequency to Loneliness in Rural Hospital Patients: A Cross-Sectional Study

**DOI:** 10.7759/cureus.59909

**Published:** 2024-05-08

**Authors:** Ryuichi Ohta, Toshihiro Yakabe, Hiroshi Adachi, Chiaki Sano

**Affiliations:** 1 Communiy Care, Unnan City Hospital, Unnan, JPN; 2 Family Medicine, Unnan City Hospital, Unnan, JPN; 3 Community Medicine Management, Shimane University Faculty of Medicine, Izumo, JPN

**Keywords:** cross-sectional studies, chronic disease, social isolation, rural population, loneliness, agriculture

## Abstract

Introduction

Agriculture is deeply woven into the fabric of rural life, influencing the economy, and the social and health dynamics of rural communities. While it offers physical and mental health benefits through regular physical activity and interaction with nature, the solitary nature of farming activities may also lead to social isolation. This study explores the complex relationship between the frequency of agricultural engagement and feelings of loneliness among rural inhabitants with chronic diseases, addressing a gap in the literature concerning the impact of agricultural practices on social well-being.

Method

A cross-sectional analysis was conducted among patients over 40 who frequented the general medicine department in Unnan City, a rural area of Japan. The study utilized the Japanese version of the University of California, Los Angeles (UCLA) Loneliness Scale to assess loneliness and collected data on agricultural activity frequency through questionnaires. Multivariate logistic regression analyses examined the association between agricultural activities and loneliness, controlling for demographic and health-related variables.

Results

Among 647 participants, higher frequencies of agricultural activities were significantly associated with increased loneliness, especially for individuals engaging in agriculture four to five times weekly or daily. Engaging in agricultural activities four to five times weekly and daily significantly increased the likelihood of higher loneliness levels, with odds ratios (OR) of 1.80 (p = 0.039) and 2.47 (p < 0.01), respectively, when compared to engagement less than once a week. Age emerged as an influential factor, with individuals aged 75 and older showing increased odds of experiencing higher loneliness (OR 1.56, p = 0.025).

Conclusion

The study underscores the dual nature of agricultural engagement in rural communities, highlighting its role in both supporting physical health and contributing to social isolation. These findings advocate for developing targeted interventions that mitigate loneliness among rural populations, suggesting the need for a balanced approach that encompasses social and healthcare strategies to enhance the overall well-being of individuals engaged in agriculture.

## Introduction

In the fabric of rural life, agriculture emerges not merely as an economic activity but as a cornerstone that sustains its inhabitants' social and health fabric [1]. This intertwining of agriculture with daily life is pivotal in shaping the lifestyles, health conditions, and social dynamics of rural communities [1-3]. Agriculture, by its very nature, involves continuous physical activity and exposure to the outdoors, which are beneficial for both physical and mental health [1]. Farming and tending to crops and livestock necessitate physical labor, which helps prevent the progression of frailty among the elderly population [4]. Moreover, the serene interaction with nature inherent in agricultural activities offers mental respite, contributing to improved mental health conditions [5]. These aspects underscore the significant potential of agriculture in supporting the well-being of rural populations.

However, the relationship between agriculture and social life in rural areas is complex and multifaceted [6,7]. While agriculture allows individuals to work independently on their lands, this solitary nature of agricultural engagement may also lead to unintended consequences of social isolation [8]. The substantial time investment required for farming activities can limit individuals' opportunities for social interaction, potentially leading to increased feelings of loneliness and social detachment [8]. This isolation can have profound implications on the mental health and overall well-being of rural inhabitants, particularly among those with existing vulnerabilities, such as chronic diseases [8].

Despite agriculture's apparent importance in rural life and its potential impact on social and health outcomes, a notable gap exists in academic research regarding the specific association between the frequency of agricultural engagement and levels of social isolation and loneliness among rural populations [2]. This gap highlights the need for targeted research to understand how agricultural practices influence the social dynamics and mental health of rural communities, especially among individuals with chronic health conditions [4].

The dual nature of agriculture as a source of physical health benefits and a potential factor for social isolation presents a complex challenge. The potential for agriculture to contribute to social isolation and loneliness in rural communities raises significant concerns, as these factors can harm mental health and overall well-being [9]. Understanding the dynamics of this relationship is crucial, particularly for individuals living with chronic diseases, who may be more susceptible to the negative impacts of isolation [10].

This research aims to delve into the intricate relationship between agricultural activity and social isolation, focusing on understanding how the frequency of agriculture influences the degree of isolation and loneliness among rural people with chronic diseases. By examining this association, the study seeks to contribute valuable insights into the complex interplay between rural lifestyles, health, and social well-being. Through this investigation, we aspire to illuminate potential pathways for enhancing the social and mental health outcomes of rural communities engaged in agriculture, thereby supporting more holistic approaches to rural health and social care.

## Materials and methods

Method

This cross-sectional study was conducted with rural citizens who regularly visited a rural Japanese community hospital to clarify the association between the frequency of agricultural activities and feelings of loneliness.

Setting

Unnan City, a distinctly rural locale in Japan, is located in the southeastern quadrant of Shimane Prefecture. As of 2020, the population of Unnan stood at 37,638, comprising 18,145 males and 19,493 females. A significant 39% of this population was over 65, a demographic expected to encompass 50% by 2025. Unnan City's healthcare facilities include 16 clinics, 12 home care stations, three visiting nurse stations, and one public hospital-Unnan City Hospital. At the time of this study, the hospital had 281 beds: 160 acute care beds, 43 comprehensive care beds, 30 rehabilitation beds, and 48 chronic care beds [11]. In addition to the clinics, the city has three visiting nurse stations and 12 home care stations. Whether working independently or through home care stations, care managers collaborate with home care patients, their families, and healthcare professionals to manage care plans and determine the necessity for professional support. Home care workers, part of these stations, assist in daily activities, providing physical care, assisted living services, and transportation [12].

Participants

All individuals over 40 who regularly attended the Department of General Medicine at Unnan City Hospital were included in the study from September 1, 2023, to November 31, 2023 [13]. Data collection involved extracting information from the electronic medical records of patients who routinely visited the hospital for management of chronic diseases or annual health checks. Furthermore, to assess the levels of loneliness and engagement in community activities, participants were administered the Japanese version of the three-item University of California, Los Angeles (UCLA) Loneliness Scale via a questionnaire [14].

Data collection

Primary Outcome

Loneliness was evaluated using the Japanese adaptation of the three-item UCLA Loneliness Scale, applicable to community-dwelling adults, with potential scores ranging from 3 to 9. This scale comprises three components: Item 1 (Companionship) asks, "How often do you feel you lack companionship?" rated on a 1-3 scale; Item 2 (Left Out) inquires, "How often do you feel left out?" with the same scaling; and Item 3 (Isolation) probes, "How often do you feel isolated from others?" also scored from 1 to 3. The total loneliness score is derived by summing the responses from these items [14].

Independent Variable

Data on the frequency of agricultural activities were gathered through a questionnaire. Participants were asked how often they engage in agricultural activities, including economic farming and recreational gardening or crop cultivation at home or in community gardens. Responses were categorized into four frequencies: less than once weekly, two to three times weekly, four to five times weekly, and more than five times weekly [13,15,16].

Covariate

Participant demographics and health data were extracted from the electronic medical records at Unnan City Hospital [13]. Collected data included age, sex, BMI for nutritional status, serum creatinine levels (mg/dL) to assess renal function, estimated glomerular filtration rate (eGFR) expressed in mL/min/1.73 m², and the Charlson comorbidity index (CCI), which evaluates the severity of various conditions such as heart failure, myocardial infarction, asthma, chronic obstructive pulmonary disease, kidney disease, liver disease, diabetes, brain stroke, brain hemorrhage, hemiplegia, connective tissue diseases, dementia, and cancer [17]. These data were updated based on the participants' most recent hospital visits for chronic conditions or annual health evaluations [13].

Statistical analysis

The Student’s t-test was used to analyze parametric data, while the Mann-Whitney U test was used for nonparametric data. Numerical variables were categorized based on the median value: scores of loneliness were divided into ≥4 (indicating higher loneliness) and <4 (indicating lower loneliness). This dichotomization was based on the close alignment of the variable's mean and median (mean: 4.17; standard deviation: 1.42; median: 4; interquartile range: 2). A univariate regression model assessed the relationship between the frequency of agricultural activities and other variables. Subsequently, a multivariate logistic regression analysis was conducted to investigate the link between the frequency of agricultural activities and increased loneliness. The multivariate logistic model included only variables that correlated with agricultural activities in the univariate analysis (p-value < 0.1). Data from participants with missing entries were excluded from the analysis. Statistical significance was established at a p-value of less than 0.05. All statistical evaluations were performed using EZR (Saitama Medical Center, Jichi Medical University, Saitama, Japan), a graphical user interface for the statistical software R (The R Foundation, Vienna, Austria) [18].

Ethical considerations

The hospital ensured the anonymity and confidentiality of the patient information used in this study. Information related to this study was posted on the hospital website without disclosing any patient details. The contact information of the hospital representative was also listed on the website to ensure that any questions regarding this study were addressed. All participants were informed of the purpose of this study and provided informed consent. The Unnan City Hospital Clinical Ethics Committee approved the study protocol (approval code: 20230010).

## Results

Participant selection

Between September 1, 2023, and November 31, 2023, 1024 patients were regularly followed by the general medicine department. Questionnaires were sent to all these patients. In total, 647 participants who answered the questionnaires were included in this study [13].

Participants’ demographics

Demographic observations revealed a significant correlation between the frequency of agricultural activities and participants' ages. Those engaged in daily agricultural endeavors were younger, with an average age of 68.16 years, compared to their counterparts participating less than once a week, who averaged 77.20 years (p < 0.001). A significant variation in gender distribution was also evident, with a higher percentage of males in the less frequent activity group (57.9%) compared to the daily activity group (39.6%, p = 0.001).

The study identified a pivotal link between the extent of agricultural engagement and the degree of loneliness, as quantified by the UCLA Loneliness Scale. Participants involved in daily agricultural activities reported a higher incidence of loneliness (58.7%) compared to those less active (38.2%, p < 0.001). Regarding the CCI, there are significant variations among the participants with different frequencies of agricultural activities (p < 0.001). Regarding specific diseases, brain hemorrhage (p = 0.047) and hypertension (p = 0.019) showed significant differences among the categorized groups. Other metrics such as BMI, eGFR, and prevalent comorbidities showed no statistically significant differences across the activity frequency spectrum (Table 1).

**Table 1:** Demographics of participants based on frequency of agricultural activities. CCI: Charlson Comorbidity Index; CKD: Chronic kidney diseases; COPD: Chronic obstructive pulmonary diseases; eGFR: Estimated glomerular filtration rate; MI: Myocardial infarction; DM: Diabetes mellitus.

Factor	Total	Less than 1 time weekly	2 to 3 times weekly	4 to 5 times weekly	Every day	P-value
N	647	76	70	169	332	
Age, mean (SD)	71.26 (12.18)	77.20 (7.66)	77.14 (6.19)	72.22 (10.01)	68.16 (13.85)	<0.001
Male sex (%)	299 (46.3)	44 (57.9)	42 (60.0)	82 (48.5)	131 (39.6)	0.001
BMI, mean (SD)	23.00 (3.81)	23.02 (2.94)	22.54 (3.22)	23.09 (3.26)	23.04 (4.33)	0.763
eGFR, mean (SD)	63.93 (15.49)	62.46 (14.68)	62.16 (14.10)	64.49 (14.43)	64.35 (16.46)	0.56
Loneliness scale, mean (SD)	337 (52.1)	29 (38.2)	24 (34.3)	89 (52.7)	195 (58.7)	<0.001
Companionship, mean (SD)	1.54 (0.63)	1.37 (0.59)	1.36 (0.51)	1.52 (0.57)	1.63 (0.67)	<0.001
Isolated, mean (SD)	1.30 (0.51)	1.22 (0.45)	1.14 (0.35)	1.24 (0.45)	1.37 (0.56)	<0.001
Left over, mean (SD)	4.17 (1.42)	3.78 (1.22)	3.70 (1.13)	4.06 (1.28)	4.41 (1.54)	<0.001
Higher loneliness (%)	337 (52.1)	29 (38.2)	24 (34.3)	89 (52.7)	195 (58.7)	<0.001
Hypertension (%)	428 (66.2)	56 (73.7)	55 (78.6)	101 (59.8)	216 (65.1)	0.019
Dyslipidemia (%)	388 (60.0)	42 (55.3)	46 (65.7)	101 (59.8)	199 (59.9)	0.644
CCI ≥ 5 (%)	218 (33.7)	32 (42.1)	31 (44.3)	52 (30.8)	103 (31.0)	0.054
CCI (%)						
0	20 (3.1)	0 (0.0)	0 (0.0)	1 (0.6)	19 (5.7)	<0.001
1	57 (8.8)	0 (0.0)	0 (0.0)	6 (3.6)	51 (15.4)	
2	82 (12.7)	9 (11.8)	2 (2.9)	28 (16.6)	43 (13.0)	
3	142 (21.9)	19 (25.0)	19 (27.1)	39 (23.1)	65 (19.6)	
4	128 (19.8)	16 (21.1)	18 (25.7)	43 (25.4)	51 (15.4)	
5	107 (16.5)	16 (21.1)	14 (20.0)	28 (16.6)	49 (14.8)	
6	64 (9.9)	11 (14.5)	10 (14.3)	17 (10.1)	26 (7.8)	
7	34 (5.3)	4 (5.3)	7 (10.0)	5 (3.0)	18 (5.4)	
8	10 (1.5)	1 (1.3)	0 (0.0)	2 (1.2)	7 (2.1)	
9	3 (0.5)	0 (0.0)	0 (0.0)	0 (0.0)	3 (0.9)	
Heart failure (%)	55 (8.5)	7 (9.2)	4 (5.7)	11 (6.5)	33 (9.9)	0.476
MI (%)	5 (0.8)	0 (0.0)	1 (1.4)	2 (1.2)	2 (0.6)	0.689
Asthma (%)	43 (6.6)	3 (3.9)	1 (1.4)	16 (9.5)	23 (6.9)	0.103
Peptic ulcer (%)	55 (8.5)	6 (7.9)	7 (10.0)	13 (7.7)	29 (8.7)	0.94
Kidney disease (%)	168 (26.0)	19 (25.0)	22 (31.4)	46 (27.2)	81 (24.4)	0.64
Liver disease (%)	52 (8.0)	4 (5.3)	5 (7.1)	16 (9.5)	27 (8.1)	0.72
COPD (%)	38 (5.9)	4 (5.3)	5 (7.1)	11 (6.5)	18 (5.4)	0.919
DM (%)	130 (20.1)	20 (26.3)	14 (20.0)	36 (21.3)	60 (18.1)	0.427
Brain infarction (%)	51 (7.9)	4 (5.3)	4 (5.7)	10 (5.9)	33 (9.9)	0.261
Brain hemorrhage (%)	13 (2.0)	4 (5.3)	1 (1.4)	0 (0.0)	8 (2.4)	0.047
Connective tissue disease (%)	85 (13.1)	8 (10.5)	9 (12.9)	25 (14.8)	43 (13.0)	0.832
Dementia (%)	12 (1.9)	0 (0.0)	2 (2.9)	0 (0.0)	10 (3.0)	0.059
Cancer (%)	69 (10.7)	10 (13.2)	11 (15.7)	14 (8.3)	34 (10.3)	0.331

Multivariate logistic regression analysis

Further analysis through multivariate logistic regression revealed a significant association between higher loneliness and the frequency of agricultural activities, as well as age. Engaging in agricultural activities four to five times weekly and daily significantly increased the likelihood of higher loneliness levels, with odds ratios of 1.80 (p = 0.039) and 2.47 (p < 0.01), respectively, compared to engagement less than once a week. Age emerged as an influential factor, with individuals aged 75 and older showing increased odds of experiencing higher loneliness (OR: 1.56, p = 0.025). Other examined variables, including male gender, a CCI of 5 or higher, and hypertension, were analyzed but did not exhibit a significant direct relationship with loneliness (p-values were 0.8, 0.1, and 0.13, respectively) (Table 2).

**Table 2:** Multivariate logistic regression model with higher loneliness and the frequency of agricultural activities. CCI: Charlson Comorbidity Index; COPD: Chronic obstructive pulmonary diseases; CI: Confidence interval.

Factor	Odds Ratio	95% CI	P-value
Agricultural activity (Less than 1 time weekly)			
2 to 3 times weekly	0.83	0.42-1.65	0.6
4 to 5 times weekly	1.80	1.03-3.16	0.039
Every day	2.47	1.46-4.19	<0.01
Age ≧ 75	1.56	1.06-2.29	0.025
Male Sex	1.04	0.75-1.44	0.8
CCI ≧ 5	0.72	0.49-1.07	0.1
Hypertension	0.77	0.54-1.08	0.13

## Discussion

The intricate interplay between agricultural activity and social isolation, particularly among individuals with chronic diseases in rural areas, remains a pivotal concern. Our research clarified that engaging in agricultural activities more than three times weekly is associated with higher conditions of loneliness among rural people, with an OR of up to 2.47. Our findings suggest a paradox within agriculture: while it harbors the potential to enhance physical health through regular physical activity and interaction with nature, it also poses a risk of increased social isolation and loneliness, especially among older populations and those with chronic health conditions.

The statistical analysis reveals a significant correlation between the frequency of agricultural activities and increased feelings of loneliness, as measured by the UCLA Loneliness Scale. Individuals engaging in agricultural activities four to five times weekly or daily exhibited higher levels of loneliness than those with less frequent engagement. This association was particularly pronounced among individuals aged 75 and older, highlighting the vulnerability of this demographic to social isolation [19-21]. These findings may indicate that engagement in frequent agricultural activities could lead to loneliness in communities due to a lack of social interaction. Alternatively, the results could suggest that people with higher levels of loneliness might engage in agricultural activities more frequently to mitigate their perception of loneliness [22]. Future studies should investigate the relationship between loneliness and the frequency of agricultural activities.

The demographic analysis within our study population underscores a broader societal issue: the aging rural demographic, with a notable portion engaged in daily agricultural activities, is at heightened risk for social isolation [22,23]. This is particularly concerning given the projection of an increasing older population within rural areas, as previous articles have shown, emphasizing the global trend of aging populations, especially in rural settings [23,24]. The implications of our findings are manifold, suggesting that while agriculture can serve as a vehicle for maintaining physical health, its role in potentially exacerbating social isolation cannot be overlooked.

On the other hand, our study does not find significant differences in loneliness levels regarding the degree of comorbidity conditions measured by CCI, indicating that the association between agricultural activity and loneliness is relatively consistent regardless of the severity of health conditions [25]. This uniformity suggests a universal aspect of the agricultural lifestyle's impact on social well-being, emphasizing the need for targeted interventions nuanced by cultural and social issues [26].

To address the complex challenges these findings pose, exploring multifaceted interventions to mitigate the risk of loneliness among rural dwellers engaged in agriculture, while respecting their cultural and social issues, is imperative. For instance, community-based programs that encourage social interaction and support networks can provide essential social contacts, potentially alleviating the loneliness experienced by this population [27,28]. However, intensive interventions requiring frequent participation may exacerbate their loneliness [29]. Integrating technology-based solutions, such as virtual communities or telehealth services, could offer additional avenues for connection, especially for those with limited mobility or access to social venues, and conditions of higher loneliness.

The synthesis of our research with existing literature elucidates a critical need for a balanced approach to rural health and social care. While agriculture remains a vital component of rural life, its potential to contribute to social isolation requires careful consideration and action [24,29]. We could not clarify the cause-and-effect relationship between loneliness and agricultural activities. Future research should identify specific relationships between them and interventions that preserve agricultural activity's health benefits and counteract its potential isolating effects [30]. Additionally, exploring the role of community structures and social policies in supporting rural populations will be crucial in developing comprehensive strategies to enhance the well-being of these communities.

One limitation of our study is its cross-sectional design, which restricts our ability to infer causal relationships between agricultural activity and loneliness. While we identified a correlation, longitudinal studies are needed to ascertain causality and the direction of this relationship. Additionally, our sample was exclusively drawn from a rural Japanese context, potentially limiting the generalizability of our findings to other rural settings with different cultural, social, and agricultural practices. The reliance on self-reported data for agricultural activity and loneliness also introduces the possibility of response bias, which may affect the accuracy of the reported associations. Future research could benefit from incorporating more objective measures of social interaction and agricultural engagement, and expanding the demographic scope to include diverse rural populations, to enhance the robustness and applicability of the findings.

## Conclusions

The study highlights the dual role of agriculture in rural Japan, sustaining physical health while also influencing social isolation among the elderly with chronic diseases. Although agricultural engagement is linked to health benefits through physical activity, our findings indicate a significant association between frequent agricultural activity and increased loneliness, particularly in older individuals. This paradox suggests that while agriculture can help maintain physical health, it may also contribute to social isolation. Addressing this issue requires innovative, culturally sensitive interventions that mitigate loneliness without undermining the health benefits of agricultural practices. Future research should focus on longitudinal studies to explore causal relationships and develop effective strategies to balance the benefits and challenges of rural agricultural lifestyles.
